# A Single-Center 18-Year Series of 73 Cases of Metaplastic Carcinoma of the Breast

**DOI:** 10.1155/2024/5920505

**Published:** 2024-01-04

**Authors:** Kassandra Thériault, Mariem Ben Moussa, Marjorie Perron, Christine Desbiens, Brigitte Poirier, Éric Poirier, Dominique Leblanc, Claudya Morin, Julie Lemieux, Jean-Charles Hogue, Dominique Boudreau

**Affiliations:** ^1^Laval University, Québec, Canada; ^2^CHU de Quebec–Laval University, 1050 Ste-Foy Road, Québec, Canada; ^3^CHU de Quebec Research Center–Laval University, 1050 Ste-Foy Road, Québec, Canada

## Abstract

**Aim:**

To examine the clinical management of metaplastic breast cancer (MeBC), particularly the role of chemotherapy.

**Methods:**

This retrospective study included patients with MeBC (*n* = 73) from a tertiary breast cancer center: the “Centre des Maladies du Sein of the CHU de Québec–Université Laval.” The specimens were reviewed by two pathologists. Patient and tumor characteristics, systemic therapy (neoadjuvant and adjuvant), disease-free survival (DFS), and overall survival (OS) were recorded.

**Results:**

The median follow-up was 57.2 months. The mean tumor size was 39.5 ± 32.1 (range, 1–200) mm. Most were in grade 3 (75.3%), without evidence of clinical nodal involvement (75.3%), and triple-negative (79.5%). Chemotherapy was given to 49 (67.1%) patients. Thirty-seven patients (50.7%) underwent a mastectomy, and 22/37 (59.5%) received radiotherapy. Adjuvant chemotherapy was given to 36 patients (49.3%), and nine (12.3%) patients were treated with neoadjuvant chemotherapy. The 5-year OS and DFS rates were 60.2% and 66.8%. Among the nine patients who received neoadjuvant chemotherapy, three (33.3%) achieved a partial response, three (33.3%) had stable disease, and three (33.3%) had disease progression. The use of chemotherapy, especially in the adjuvant setting, had a significant positive effect on 5-year OS (*P*=0.003) and 5-year DFS (*P*=0.004). Nodal involvement was associated with worse OS (*P*=0.049) but similar DFS (*P*=0.157). Lumpectomy was associated with better 5-year OS (*P* < 0.0001) and DFS (*P*=0.0002) compared with mastectomy.

**Conclusion:**

MeBC represents a rare heterogeneous group of malignancies with poor prognosis. Adjuvant chemotherapy was associated with improved OS and DFS. Patients should be carefully selected for neoadjuvant chemotherapy.

## 1. Introduction

Breast cancer is responsible for 11.6% of the new cases of cancer each year and 6.6% of cancer-related mortality worldwide [1]. Metaplastic breast cancer (MeBC) represents a rare heterogeneous group of malignancies comprising 0.2%–5% of all invasive breast cancers [2].

The current World Health Organization classification of MeBC includes low-grade adenosquamous carcinoma, fibromatosis-like metaplastic carcinoma, squamous cell carcinoma, spindle cell carcinoma, carcinoma with mesenchymal differentiation, and mixed MeBC [3, 4]. Except for low-grade adenosquamous carcinoma and fibromatosis-like variants, MeBCs are typically aggressive, resistant to chemotherapy, and have a greater propensity for metastases than nonmetaplastic breast tumors [5–8]. They often present with a triple-negative phenotype, a high tumor stage, and a high tumor grade. These tumors arbor unique pathological features and, currently, the molecular drivers for these tumors are not entirely understood. Previous studies showed that MeBCs are more aggressive than triple-negative breast cancers in terms of disease-free survival (DFS) and overall survival (OS) [5–8].

The standard treatment for most MeBCs includes surgery ± radiation therapy, but the use of chemotherapy is based on clinical trials involving typical triple-negative invasive breast cancers. Patients with MeBCs often receive chemotherapy, reaching 73% in retrospective cohorts, but it is known that MeBCs are relatively chemoresistant [9–11]. In addition, they tend to have a low rate of pathological complete response (pCR) to neoadjuvant chemotherapy [11].

Despite the recognition of the MeBC entity in the early 2000s, there is a lack of evidence on managing patients with MeBC because they are treated as conventional invasive ductal carcinoma (IDC). Therefore, this study aimed to examine the clinical management of MeBC, particularly the role of chemotherapy. The results could help improve the management of patients with MeBC.

## 2. Materials and Methods

### 2.1. Study Design and Patients

At the Center des Maladies du Sein of the CHU de Québec–Université Laval, all breast cancer patients are prospectively compiled in a cancer registry by oncology registrars or nurses trained in oncology. This retrospective study included patients diagnosed with MeBC from January 2004 to November 2020. Patients were included if they were diagnosed and treated at our center. All specimens and slides of the identified patients were reviewed by two breast pathologists. Each tumor was reclassified according to the recent World Health Organization classification WHO [3, 4]. Tumors were considered pure if the morphology was found in more than 90% of the tumor. If more than one histologic subtype was found or an invasive carcinoma NOS was admixed, the tumor was classified as mixed metaplastic carcinoma. Three patients were excluded because of the absence of histological slides or diagnostic changes after review. This study was approved by the ethics committee of the CHU de Québec–Université Laval (#2021–5649). The requirement for individual informed consent was waived by the committee because of the retrospective nature of the study.

### 2.2. Data Collection

The following clinicopathologic data were recorded: age at diagnosis, TNM, biomarkers (estrogen receptors (ER), progesterone receptor (PR), and HER2), locoregional treatment (surgery and radiation therapy), systemic therapy (neoadjuvant and adjuvant), and survival data, including disease-free survival (DFS) and OS. DFS was defined as the time from diagnosis to developing any recurrence (distant or locoregional) or death. OS was defined as the time from diagnosis to death from any cause. In the absence of a DFS or OS event, survival was censored at the last follow-up.

### 2.3. Statistical Analysis

Descriptive statistics were used. Continuous data were presented as median (range). Categorical data were presented as *n* (%). Survival was analyzed using the Kaplan–Meier method, and differences among characteristics were tested using the log-rank test. All analyses were conducted using SAS 9.4 (SAS Institute, Cary, NC, USA). Two-sided *P* values <0.05 were considered statistically significant.

## 3. Results

### 3.1. Characteristics of the Patients

Seventy-six cases were reported as MeBC in the cancer registry, but the pathological slide review confirmed 73 cases. The patient and tumor characteristics are detailed in Table 1. The study included 29 patients (39.7%) with mixed metaplastic carcinoma, 18 (24.7%) with metaplastic carcinoma with heterologous mesenchymal differentiation, 13 (17.8%) with squamous cell carcinoma, 11 (15.1%) with spindle cell carcinoma, one (1.4%) with low-grade adenosquamous carcinoma, and one (1.4%) with fibromatosis-like metaplastic carcinoma. The median age at diagnosis was 61.5 years (range, 32–96 years). Fifty-six (76.7%) patients were menopausal at diagnosis.

The mean tumor size was 39.5 ± 32.1 (range, 1–200) mm. Most were triple-negative (79.5%). ER-positive and PR-positive MeBCs were found in 12.3% and 5.5% of the patients, respectively, and 4.1% were HER2-positive. Most MeBCs showed grade 3 nuclear grade (75.3%) and were pathologically node-negative (pN0) (65.8%). Lymphovascular invasion was present in 13 (17.8%) patients. There were 12 patients (16.4%) with clinical nodal involvement at presentation (cN+), and five patients (6.8%) were diagnosed with de novo metastatic disease.

Nine (12.3%) received neoadjuvant chemotherapy. Half the patients (49.3%) underwent a lumpectomy, while 50.7% underwent a mastectomy. Sentinel lymph node biopsy was more frequent (53.5%) than axillary dissection (35.6%). Regarding systemic treatments, chemotherapy alone was offered to 42 patients (57.5%), and seven (9.6%) received both chemotherapy and hormonal therapy. Adjuvant systemic therapy was administered to 36 patients (49.3%). Finally, 53 (72.6%) patients were treated with adjuvant radiation therapy; 33 of the 36 patients who underwent lumpectomy also received radiation therapy (Table 1).

### 3.2. Neoadjuvant Chemotherapy Subgroup

While most patients were treated with adjuvant chemotherapy and radiation therapy, nine with a locoregional disease received neoadjuvant chemotherapy (Table 2). Six patients had a cT3 tumor, and two had a cT4 tumor. After preoperative chemotherapy, three patients (33.3%) achieved a clinical partial response, three patients (33.3%) had stable disease, and three patients (33.3%) had disease progression. Despite neoadjuvant chemotherapy, most patients underwent a total mastectomy (77.8%). One (11.1%) patient had a pCR.

### 3.3. Survival

**Figure 1:**
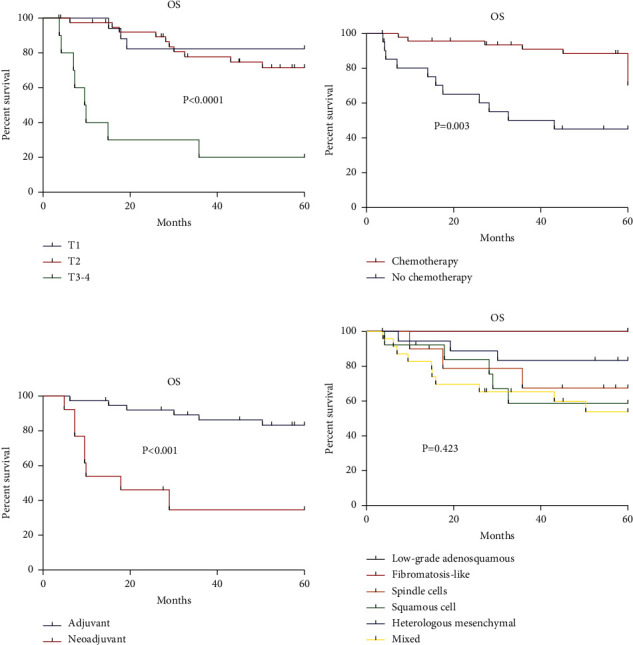
Kaplan–Meier curves of overall survival according to (a) *T* stage, (b) chemotherapy, (c) type of chemotherapy, and (d) pathological subtype.

Survival analyses were done excluding the de novo metastatic patients (*n* = 5). The average follow-up was 57.2 months. The 5-year OS and DFS rates were 66.8% (Table 3) and 60.2% (Table 4), respectively. The OS and DFS varied among histological subtypes, with low-grade adenosquamous carcinoma and fibromatous-like metaplastic carcinoma histologic showing better outcomes. In the univariable analyses, a higher *T* stage (*P* < 0.0001), pathological nodal involvement (*P*=0.049), no chemotherapy use (*P*=0.003), the use of neoadjuvant chemotherapy as opposed to adjuvant chemotherapy (82.8% vs. 25.0%; *P*=0.001), and mastectomy (*P* < 0.0001) were associated with poorer OS (Table 3 and Figure 1). Higher clinical tumor stage (*P*=0.0002), the use of chemotherapy (*P*=0.004), neoadjuvant chemotherapy (*P*=0.001), and mastectomy (*P*=0.0002) were associated with poorer DFS (Table 4). Radiation therapy did not influence OS (*P*=0.107) or DFS (*P*=0.191). Multivariable analyses were not possible due to the small sample size.

## 4. Discussion

Because of the rarity of MeBCs, the knowledge about the treatment patterns and outcomes of these tumors is limited, and data are missing on the optimal management of this historically known aggressive type of breast cancer. This single-center retrospective study of 73 patients with MeBC evaluated the correlation between clinicopathological features and the choice of therapy on survival outcomes.

MeBCs appear to have multiple clinicopathologic parameters that differentiate them from other types of breast cancer. Compared with IDC, they tend to occur in older women and present with larger tumor sizes, reflecting a more rapid growth rate [7, 9, 12–14]. In the present study, patients with a locoregional disease (*n* = 68) had a 5-year DFS and OS of 60.2% and 66.8%, respectively. These results were consistent with previous retrospective studies that showed worse OS for MeBCs than other breast cancer subtypes [7, 8, 14, 15].

Data are conflicting about the effectiveness of chemotherapy for MeBC [7, 14, 16]. A review from Tzanninis et al. [17] comprising 12 studies did not demonstrate an OS benefit with neoadjuvant or adjuvant chemotherapy. On the other side, a contemporary large retrospective study using the National Cancer Database (NCDB) compared 5142 MeBC with 50,705 TNBC cases and found that the omission of chemotherapy led to worse OS (HR = 1.527; *P*=0.007) [8]. Accordingly, our results showed that omission of chemotherapy was associated with worse 5-year DFS (*P*=0.004) and OS (*P*=0.003). Therefore, controversy remains about the real effectiveness of chemotherapy in patients with MeBC, although often it is still administered given the triple-negative and aggressive nature of this cancer.

We reported nine (11.5%) patients who underwent neoadjuvant chemotherapy. Three patients had stable disease (33.3%), three had a partial clinical response (33.3%), and three (33.3%) had progressive disease while on neoadjuvant chemotherapy. Despite receiving neoadjuvant therapy, most patients underwent a total mastectomy (77.8%), reflecting a low conversion rate to partial mastectomy. These results confirm those of the literature. A recent report from the Mayo Clinic Rochester, including 18 patients with MeBC receiving neoadjuvant chemotherapy, showed that five (27.8%) patients progressed on treatment, including two who became metastatic [18]. Wong et al. [19] identified 44 patients with MeBC treated with neoadjuvant chemotherapy, among whom 49% showed stable or progressive disease. In addition, our results demonstrate a lower OS among patients treated with neoadjuvant chemotherapy compared to adjuvant treatment (25.0% vs. 82.8%, *P*=0.001). It could be explained by the fact that patients treated with neoadjuvant have larger tumors, which is an important prognostic factor, sometimes combined with other factors of poor prognosis. Based on these results and previous studies, we might hypothesize that upfront surgery is safer for patients with an initially operable tumor, followed by systemic therapy. Multicenter studies are necessary to examine that hypothesis.

Some metaplastic carcinoma subtypes have better survival than others and possibly different susceptibilities to chemotherapy [11]. Previous studies showed that low-grade adenosquamous carcinoma and fibromatosis-like metaplastic carcinoma histologic subtypes have a relatively good prognosis [3]. Although the present study included a few patients, a 100% 5-year OS was also observed among these two subtypes, and the association between the histological subtypes and OS was statistically nonsignificant (*P*=0.423). On the other hand, some subtypes, such as high-grade spindle cell carcinoma, carcinoma with pleomorphic components, and squamous cell carcinoma, show a higher propensity for metastases [20].

Consistent with previous reports, the results reported here showed that a higher clinical *T* stage (cT3-T4 vs. cT1-T2) significantly predicted poorer DFS and OS [14]. It could explain the worse survival observed among patients who underwent a total mastectomy, with a 5-year OS of 43.5%, compared with 90.3% among patients who underwent breast-conserving surgery (*P* < 0.0001) in the present study.

As with stage *T*, lymph node status is also an important prognostic factor. Although metaplastic carcinoma gives rise to lymph node metastases less often than intraductal invasive cancers [8, 9, 13], lymph node involvement is an independent prognostic factor [12, 13, 15, 21], as supported by the present study. Most of our patients (*n* = 60, 76.1%) had no lymph node involvement, and 53.4% underwent sentinel lymph node biopsy, but the prognosis of those with lymph node involvement remained poor, showing a 5-year OS of 42.9% vs. 73.5% (*P*=0.036) among patients without node metastases.

The available therapies seem to improve the poor prognostic of MeBCs, but still, novel treatments are needed. MeBCs are characteristically triple-negative, eliminating the options of hormone and anti-HER2 therapies. Additional analyses of these tumors have pointed out molecular and genomic alterations that could be potential therapeutic targets. In this way, MeBCs are frequently associated with mutations in TP53 (26%–75%) and PIK3CA (23%–70%) [22]. Other mutations identified include PTEN, NF1, HRAS, and PIK3R1. These mutations could lead to future studies evaluating the effectiveness of targeted treatments.

Pivotal trials demonstrated the oncological equivalence of lumpectomy with radiation therapy compared with mastectomy [23, 24], but a recent study of 48,986 Swedish women showed that lumpectomy with radiation therapy achieved better survival than mastectomy, irrespective of radiation therapy [25]. Population-based studies also showed better OS after lumpectomy with radiation therapy compared with mastectomy [26–29]. The change in paradigm between the original pivotal trials [23, 24] and the recent ones [25–29] could be due to several factors, including improvements in systemic therapies and radiation therapy. Furthermore, studies also showed that mastectomy has no survival benefit over lumpectomy with radiation therapy in younger patients and in those with triple-negative breast cancer [30–32]. A study in MeBC also reported a higher (but not statistically significant) 5-year PFS for lumpectomy compared with mastectomy (69% vs. 61%, *P*=0.22) [33], supporting the present study. Still, the differences between lumpectomy and mastectomy were important in the present study (OS: 90.3% vs. 43.5%; DFS: 81.4% vs. 38.1%). The reasons for the differences could not be determined based on the present study.

This study has several limitations. It is a retrospective single-center cohort study, and the sample size is small. As a retrospective study, treatment characteristics were missing, the patients were treated in different manners, some patients received older systemic regimens, and some data were missing for some patients. Although all patients were discussed in tumor boards, the discussions and rationale for the final treatment decision were not indicated in the charts. In addition, as a result of the small cohort, it was impossible to perform multivariable analyses because of the few events (recurrence and death) in specific subgroups. The population was predominantly white and non-Hispanic and the results might not reflect other populations. Data about follow-up were only available for the consultations at the CHU de Québec–Université Laval, and patients who received treatment outside our institution may not have been documented.

In conclusion, MeBCs represent a rare, heterogeneous group of malignancies with poor outcomes, even worse than TNBC. The literature about MeBCs suggests benefits for chemotherapy for a disease that we thought was relatively chemoresistant. Still, previous studies and the present one revealed a significant rate of progression on neoadjuvant chemotherapy, suggesting that upfront surgery followed by systemic therapy might be safer for patients with an initially operable tumor, but that point must be confirmed in large-scale multicenter studies.

## Figures and Tables

**Table 1:** Characteristics of the patients (*n* = 73).

Age (years)	
≤55	26 (35.6%)
>55	47 (64.4%)
Median (range)	61.5 (32–96)
Menopausal	56 (76.7%)
Subtype	
Adenosquamous, low-grade	1 (1.4%)
Fibromatosis-like	1 (1.4%)
Spindle cell	11 (15.1%)
Squamous cell	13 (17.8%)
Heterologous mesenchymal cell differentiation	18 (24.7%)
Mixed	29 (39.7%)
Clinical stage	
cT	
cT1	16 (21.9%)
cT2	26 (35.9%)
cT3	16 (21.9%)
cT4	7 (9.6%)
Unknown	8 (11.0%)
cN	
cNx	6 (8.2%)
cN0	55 (75.3%)
cN1	10 (13.7%)
cN2	2 (2.7%)
cN3	0
cM	
cMx	3 (4.1%)
cM0	65 (89.0%)
cM1	5 (6.9%)
Nuclear grade	
G1	1 (1.4%)
G2	7 (9.6%)
G3	55 (75.3%)
Unknown	10 (13.7%)
Lymphovascular invasion	13 (17.8%)
HER2—positive	3 (4.1%)
HER2—unknown	4 (5.5%)
Combined receptor profile	
HR+, HER2+	1 (1.4%)
HR−, HER2−	58 (79.5%)
HR+, HER2−	8 (11.0%)
HR−, HER2+	2 (2.7%)
HR+, HER2?	3 (4.1%)
HR−, HER2?	1 (1.4%)
Surgery	
Breast-conserving surgery	36 (49.3%)
Mastectomy	37 (50.7%)
Axillary surgery	
Sentinel lymph node biopsy	39 (53.4%)
Axillary dissection	26 (35.6%)
None	8 (11.0%)
Radiation therapy	53 (72.6%)
Treatments	
Breast-conserving surgery, RT	33 (45.2%)
Breast-conserving surgery, no RT	3 (4.1%)
Mastectomy, RT	20 (27.4%)
Mastectomy, no RT	17 (23.3%)
Systemic therapy	51 (69.9%)
Chemotherapy	42 (57.5%)
Chemotherapy and hormonal therapy	7 (9.6%)
Hormonal therapy	2 (2.7%)
Systemic chemotherapy (*n* = 49)	
Cyclophosphamide and doxorubicin	11 (22.5%)
Cyclophosphamide and epirubicin	1 (2.0%)
Docetaxel	1 (2.0%)
5-fluorouracil, doxorubicin, and cyclophosphamide	1 (2.0%)
5-fluorouracil, doxorubicin, cyclophosphamide, and taxane	8 (16.3%)
Cyclophosphamide, doxorubicin, and taxane	5 (10.2%)
Carboplatin and taxane	2 (4.1%)
Carboplatin and cyclophosphamide	8 (16.3%)
Docetaxel, doxorubicin, and cyclophosphamide	1 (2.0%)
Clinical trial	4 (8.2%)
Unknown	7 (14.3%)
Trastuzumab	2 (2.7%)
Chemotherapy	49 (49.7%)
Adjuvant	36 (49.3%)
Neoadjuvant	9 (12.4%)
Metastatic	4 (5.5%)
Pathological stage pT	
pTx	1 (1.4%)
pT0	1 (1.4%)
pT1	16 (21.9%)
pT2	42 (57.5%)
pT3	9 (12.3%)
pT4	4 (5.5%)
pN	
pNx	8 (11.0%)
pN0	48 (65.8%)
pN1	9 (12.3%)
pN2	4 (5.5%)
pN3	4 (5.5%)

HR: hormonal receptor; LN: lymph node; RT: radiotherapy.

**Table 2:** Characteristics of the patients treated with neoadjuvant chemotherapy (*n* = 9).

Age (years)	
≤55	4 (44.4%)
>55	5 (55.6%)
Median (range)	58 (32–66)
Subtype	
Mixed	3 (33.3%)
Metaplastic only	6 (66.7%)
Main component	
Matrix-producing	1 (11.1%)
Squamous	1 (11.1%)
Mixed	2 (22.2%)
Malpighian	2 (22.2%)
Sarcomatous	1 (11.1%)
Fusiform	2 (22.2%)
Estrogen receptors—positive	2 (22.2%)
Progesterone receptors—positive	0
HER2—positive	0
Clinical stage pretreatment	
cT	
cT2	1 (11.1%)
cT3	6 (66.7%)
cT4	2 (22.2%)
cN	
cN0	3 (33.3%)
cN1	3 (33.3%)
cN2	1 (11.1%)
Unknown	2 (22.2%)
cM	
cM0	8 (88.9%)
cM1	1 (11.1%)
Surgery	
Breast-conserving surgery	2 (22.2%)
Mastectomy	7 (77.8%)
Complete pathological response	1 (11.1%)

**Table 3:** Survival analyses, overall survival (OS).

	Survival (%)	*P*
5-year OS rate	66.8	—
Pathological T		<0.0001
pT0-1	82.4	
pT2	71.5	
pT3-4	20.0	
Pathological N		0.049
pN0	73.5	
pN+	42.9	
Chemotherapy		0.003
Yes	73.5	
No	50.2	
Chemotherapy type		0.001
Adjuvant	82.8	
Neoadjuvant	25.0	
Radiotherapy		0.107
Yes	72.4	
No	51.3	
Surgery type		<0.0001
Mastectomy	43.5	
Breast-conserving surgery	90.3	
Pathological subtype		0.423
Adenosquamous, low-grade	100	
Fibromatosis-like	100	
Spindle cell	67.5	
Squamous cell	58.7	
Heterologous mesenchymal cell differentiation	83.3	
Mixed	53.9	
Pathological subtype, grouped		0.094
Nonmixed	73.4	
Mixed	53.9	

**Table 4:** Survival analyses, disease-free survival (DFS).

	DFS (%)	*P*
5-year DFS rate	60.2	—
Pathological T		0.0002
pT1	70.6	
pT2	65.5	
pT3-4	20.0	
Pathological N		0.157
pN0	64.4	
pN+	42.9	
Chemotherapy		0.004
Yes	70.2	
No	35.3	
Chemotherapy type		0.001
Adjuvant	77.0	
Neoadjuvant	50.0	
Radiotherapy		0.191
Yes	65.1	
No	45.7	
Surgery type		0.0002
Mastectomy	38.1	
Breast-conserving surgery	81.4	
Pathological subtype, grouped		0.459
Nonmixed	66.9	
Mixed	48.2	

## Data Availability

The data used to support the findings of this study are available from the corresponding author upon reasonable request.
